# Development and preliminary evaluation of the Chinese adult dietary behavior scale

**DOI:** 10.3389/fnut.2025.1647600

**Published:** 2025-10-23

**Authors:** Haiyue Zhang, Xinrui Li, Yuan Wang, Wei Zhang, Ying Liang, Yue Wang, Zhe Yang, Zhijun Tan, Junrong Xu, Lei Shang

**Affiliations:** ^1^Ministry of Education Key Lab of Hazard Assessment and Control in Special Operational Environment, Department of Health Statistics, School of Public Health, Fourth Military Medical University, Xi’an, Shaanxi, China; ^2^School of Public Health, Xi’an Medical College, Xi’an, Shaanxi, China; ^3^Department of Gastroenterology, YuLin No.4 Hospital, Yulin, Shaanxi, China; ^4^Department of Gastroenterology, Xi'an No.3 Hospital, The Affiliated Hospital of Northwest University, Xi'an, Shaanxi, China

**Keywords:** dietary behavior, scale, Chinese adults, reliability, validity

## Abstract

**Background:**

There is currently no widely accepted multidimensional tool to assess adult dietary behaviors in China. This study developed the Chinese Adults Dietary Behavior Scale (CADBS) to evaluate Chinese adults’ eating-related traits and preliminarily examined its reliability, validity, and ability to distinguish different groups.

**Methods:**

The scale was developed through three rounds of surveys conducted from 2020 to 2021. Items were screened using item analysis. After item selection, exploratory and confirmatory factor analyses were used to determine the final structure of the scale, and its reliability, structural validity, and discriminative ability were assessed accordingly.

**Results:**

The final scale consisted of 7 dimensions with 39 items: snacking, food responsiveness, emotional eating, restrictive eating, food fussiness, healthy dietary awareness, and external eating. Exploratory factor analysis showed a total cumulative variance contribution rate of 60.60%. Confirmatory factor analysis yielded a Tucker–Lewis index (TLI) of 0.892 and a comparative fit index (CFI) of 0.903. The total scale had a Cronbach’s *α* coefficient of 0.890, a split-half reliability coefficient of 0.920, and a test–retest reliability coefficient of 0.740. Snacking (*β* = 0.422), food responsiveness (*β* = 0.412), and restrictive eating (*β* = 0.675) were positively associated with BMI, while healthy dietary awareness (*β* = −0.396) was negatively associated with BMI.

**Conclusion:**

Following standard procedures, this study developed a self-report scale for assessing Chinese adults’ dietary behaviors. The CADBS has good reliability and structural validity, making it suitable for epidemiological surveys of dietary behaviors and public health intervention practices. Specifically, it shows that Chinese adults who snack more have stronger food responsiveness and are more likely to be overweight or obese. Conversely, an overweight or obese Chinese adult tends to be restrictive about eating. On the contrary, those with greater healthy dietary awareness are more likely to have a normal weight.

## 1 Introduction

Dietary behavior refers to the quantity, type, and patterns of food intake, serving as a foundational determinant of weight management and overall health. It is directly relevant to the global burden of overweight and obesity and the risk of diseases such as diabetes and cardiovascular disorders (1). Unhealthy dietary behaviors, including emotional eating, rapid eating, and excessive responsiveness to food cravings, act as key drivers of these health issues, alongside genetic and environmental factors (2–6). Therefore, the early identification and timely correction of these behaviors are crucial for mitigating health risks, establishing an accurate dietary assessment as a cornerstone of global obesity prevention and chronic disease management. This global relevance is particularly salient in China, where rapid socioeconomic progress has dramatically altered dietary patterns and increased the prevalence of high-calorie food consumption (7).

China faces a severe and escalating overweight and obesity crisis. Globally, obesity rates have nearly tripled since 1975 (8). Within China, the 2020 Report on Chronic Diseases and Nutrition Among Chinese Residents indicates that over half of adults are overweight or obese, with 34.3% classified as overweight and 16.4% as obese (≥18 years) (9–11). Projections suggest that by 2030, obesity will affect 21% of adult women and 29% of adult men (9, 12). These alarming trends are placing increasing strain on public health systems and highlight the urgent need for effective tools to assess and modify dietary behaviors.

Several validated and reliable questionnaires have been widely adopted globally, such as the Dutch Eating Behavior Questionnaire (DEBQ) (13), the Three-Factor Eating Questionnaire (TFEQ) (14), and the Adult Eating Behavior Questionnaire (AEBQ) (15). Each serves distinct purposes. The TFEQ focuses on Cognitive Restraint, Disinhibition, and Hunger. The DEBQ assesses emotional eating, restraint, and external eating. The AEBQ, adapted from the Child Eating Behavior Questionnaire, examines a wider range of appetitive traits, including hunger, food responsiveness, emotional over-eating, enjoyment of food, satiety responsiveness, emotional under-eating, food fussiness, and slowness in eating. Although these instruments have proven valuable in Western contexts, their applicability to the Chinese population remains limited. This limitation arises primarily because they were designed around Western dietary patterns and cultural norms. Consequently, they fail to capture key constructs specific to Chinese dietary habits, including the increasing influence of fast food and snacks (16), risks inherent to traditional practices such as frequent frying, and a cultural emphasis on taste that often overshadows nutritional considerations.

China’s unique food culture and recent dietary transitions further justify the need for a culturally tailored assessment tool. National survey data, such as the 2015 China National Nutrition and Health Survey (CNNHS 2015), highlight key shifts in Chinese dietary behavior: increased snack consumption, changes in cooking practices, and widespread meal skipping, particularly breakfast (17, 18). Over the past 15 years, snacking—defined as consuming foods or beverages outside breakfast, lunch, and dinner (19–21)—has often replaced main meals among young adults, increasing vulnerability to nutritional imbalance. Most snacks are high in sugar, salt, and fat, elevating the risk of overweight, obesity, and hyperglycemia (22, 23). Concurrently, China’s dietary transition includes a surge in animal product consumption, alongside increased intake of cooking oils, salt, and added sugars, despite persistently low intake of essential foods such as milk, dairy products, and vitamins (24). Large prospective cohort studies further suggest that greater dietary variety, better nutritional balance, and higher fruit and egg intake are associated with reduced all-cause mortality (25). These dietary patterns emphasize that international tools cannot fully quantify China-specific dietary behaviors or associated health risks.

Against this backdrop, a standardized, culturally tailored tool for assessing Chinese adults’ dietary behavior is still lacking. To our knowledge, no such instrument has been fully developed and validated to address China’s unique dietary context. The Healthy China Initiative has raised public awareness of health issues related to overweight and obesity (26), creating a critical demand for evidence-based tools to inform prevention and nutritional intervention strategies. To fill this void, the present study aimed to develop and validate the Chinese Adults Dietary Behavior Scale (CADBS)—a reliable, valid, and effective tool for assessing the appetitive characteristics of Chinese adults. This scale will provide a robust measurement framework to support targeted public health efforts and contribute to improved management outcomes for nutrition-related chronic diseases in China.

## 2 Materials and methods

### 2.1 Participants

**Figure 1:**
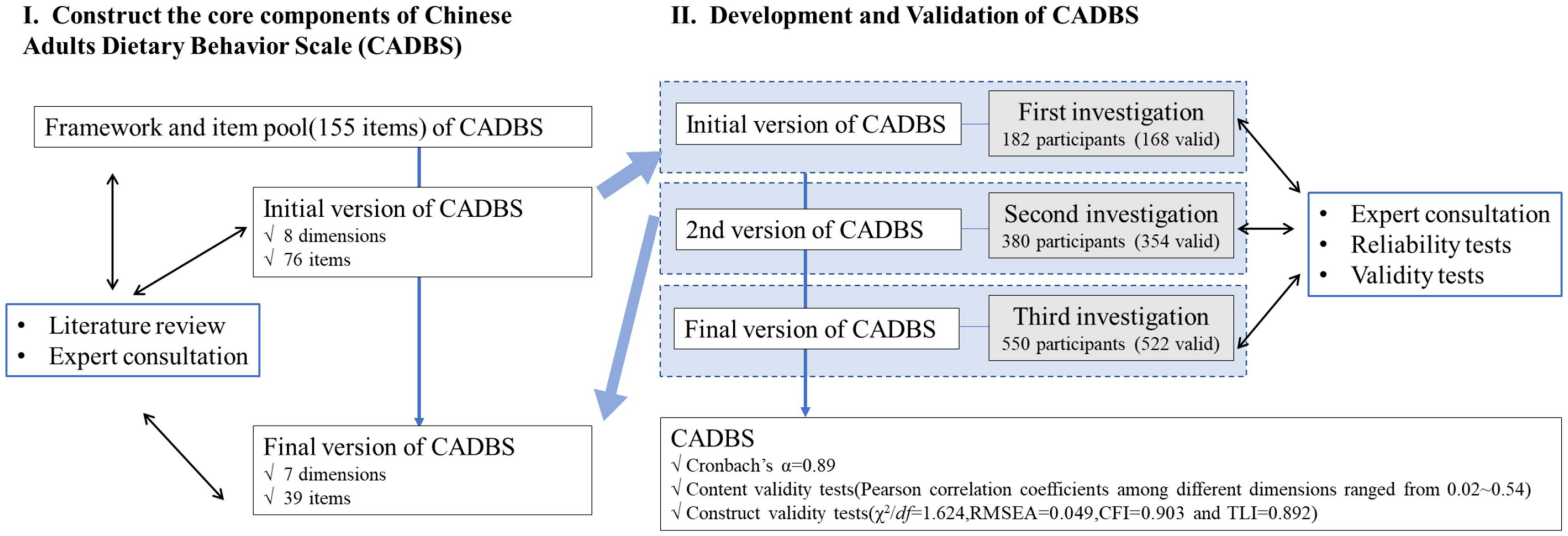
Study flow diagram of the whole process of development and preliminary evaluation of the Chinese Adults Dietary Behavior Scale (CADBS).

To ensure a comprehensive assessment of reliability parameters, a total of 522 adults were recruited for this study. The sample size was determined based on the widely recognized guideline of including at least 10 participants per questionnaire item (27). The study enrolled healthy adult volunteers (aged 18 years or older) who resided in the city of Xi’an, China, between 2020 and 2021. To obtain a representative sample of adults from varying locations, researchers recruited participants from different communities in Xi’an as well as from a large general hospital’s physical examination center. The workflow of CADBS development and validation is shown in Figure 1.

The inclusion criteria for the questionnaire were Chinese adults over 18 years, excluding those having chronic digestive diseases, diabetes, malignant tumors, hyperthyroidism, and other conditions, which may affect appetite and eating, as well as women during pregnancy and lactation. Adults who were illiterate or reluctant to participate were also excluded from the study. Demographic variables (age and gender) and anthropometric variables (weight and height) were included in the CADBS. BMI was calculated using body weight in kilograms divided by the square of height in meters. BMI is a universal index used to define overweight and obesity in accordance with standards as follows: underweight: < 18.5 kg/m^2^, normal weight: 18.5–23.9 kg/m^2^, overweight: 24–27.9 kg/m^2^, and obesity: ≥ 28 kg/m^2^ (28). Only participants who answered all items of the questionnaire were included in the analysis afterwards.

The study protocol was approved by the Institutional Ethics Committee of Xi’an Third Hospital (Approval No: SY11-2020-086). Written informed consent was obtained from all participants prior to their inclusion in the study.

### 2.2 Conceptual model selection and development of the draft version of the CADBS

A conceptual framework was constructed to define the dimensionality of the CADBS. A literature review was conducted on both international literature and literature written in Chinese. In addition to that, two qualitative interviews were carried out to supplement the literature review. The qualitative interviews were conducted in three steps: first, a face-to-face meeting was held to discuss and collect sufficient information on adults’ dietary behavior in-depth. Next, a qualitative consensus study was conducted to determine the dimensions and core components of adults’ dietary behavior. Finally, an expert discussion meeting was held to discuss, analyze, and modify the core components of Chinese adults’ dietary behavior in consideration of its scientificity, rationality, applicability, and readability. Moreover, Chinese food and dietary culture (e.g., concerns about healthy eating and nourishment, eating habits that focus on taste rather than the nutritional value of food) were also considered in the conceptual framework. As a result, an eight-dimensional conceptual framework was developed to comprehend the dietary behavior of Chinese adults. Based on the conceptual framework, a pool of 155 items (i.e., questions) was initialized to measure the core components of the Chinese Adults Dietary Behavior Scale. A list of validated instruments was referenced during the development of the 155 items, including DEBQ (13), TFBQ (14), AEBQ (15), EBS (29), and EAT (30).

These referenced instruments were then translated into Chinese in two steps: forward translation and backward translation. The forward translation of the questionnaire was carried out by a clinical nutritionist with the help of a professional translator. Backward translation was carried out by an independent native English speaker who is fluent in Chinese and medical terminology. A critical review was performed to compare the backward translation with the original version. Linguistic errors and terms that may need adjustments were discussed to decide whether to change or keep them due to language/cultural differences. Afterward, items were evaluated in terms of their semantic content by experts in the fields of gastroenterology, nutrition, and statistics. Words and terms reported as confusing or difficult to recognize were deleted. Finally, 76 items were retained to constitute the initial version of the CADBS, and it was approved for pilot testing.

### 2.3 Scoring methods

Each item in the questionnaire measures the frequency of dietary behavior that survey participants experienced within 2 weeks. The Likert-type scale was used to rate each item of the questionnaire (from 1 = never to 5 = always). The dimensional score was the average of the items from the corresponding dimension. A higher score indicates that respondents are inclined towards the behavior that the item implied.

### 2.4 Investigation methods

#### 2.4.1 Development of the trial questionnaire (for the first investigation)

As stated above, the initial version of the CADBS contains a 76-item questionnaire in accordance with eight dimensions. The initial version of the questionnaire was given out to 182 adults in Xi’an through the internet. Inclusion and exclusion criteria were applied. All participants completed the first draft of the CADBS independently, and 168 participants returned the questionnaire to the investigators. Item analysis and construct validity of the first version of the questionnaire were assessed to determine reliability and validity.

#### 2.4.2 Development of the final questionnaire (for the second investigation)

After the first investigation, 47 items in accordance with eight dimensions were retained. Moreover, redundant components were eliminated or merged. For example, the items “I eat less when I feel sad” and “I eat less when I feel down” were merged into “I eat less when I’m upset.” A sample of 380 questionnaires was given out, and 354 of them were returned. Next, the second version of CADBS was gone through for item analysis and construct validity to make it more understandable and accurate for the meaning conveyed. In addition, minor adjustments to the wording of some questions were made. At the end, 8 items were eliminated, and the second version of CADBS consisted of 7 dimensions with 39 items, which was the final questionnaire.

#### 2.4.3 Evaluating the final questionnaire (for the third investigation)

The questionnaire was applied to a sample of 550 individuals in three communities from both urban and suburban areas of Xi’an, and 522 of them were returned. The goal of the third investigation was to evaluate the factor structure and test the robustness and meaningfulness of the finalized items. To conduct test–retest validity, a subsample of 80 adults was asked to answer the questionnaire 2 weeks later.

### 2.5 Item analysis

Item analysis explains how well a set of items can measure one characteristic (or construct), which typically reflects the questionnaire’s reliability and discrimination. Statistics are involved in item analysis, such as item mean, standard deviation, corrected item-total correlation, reliability if the item was deleted, and the alpha reliability of the scale. Items went through item selection by five criteria, and items retained should meet at least three out of the five criteria (31). The 5-item selection criteria can be described as follows: (1). Standard deviation of an item < 0.85 was not the criterion; (2). The critical ratio analysis method: The total score was obtained by adding up all item scores. The cutoff value was set at 27% of the total score; the top and bottom 27% of the population were identified as two groups of participants. An independent sample t-test between the two groups was conducted for each item, and those with a *p* value of > 0.05 did not meet the criterion; (3). Principal components analysis (PCA) was conducted to calculate item loadings. Items with a factor loading of < 0.4 did not meet the criterion; (4). Cronbach’s alpha coefficients were calculated for each item. The term of reliability was used if the item deleted was checked, and if this term improves, the corresponding item did not meet the criterion; and (5). Pearson’s correlation coefficient was calculated between each item and the total score, and items with a corrected item-total correlation (CITC) of < 0.4 did not meet the criteria.

### 2.6 Reliability analysis

To conduct a reliability analysis of a questionnaire, the sample size should be at least 5–10 times the number of items. The CADBS contains 8 dimensions with 39 items, which means that at least 195 participants were required to obtain an adequate sample size for the reliability analysis. Reliability was assessed by evaluating internal consistency and test–retest. Cronbach’s alpha coefficient was used to evaluate internal consistency, and a coefficient of > 0.7 can be considered the questionnaire having good reliability (the minimum shall not be less than 0.6) (32, 33). To conduct test–retest, participants who had already filled out the self-administered questionnaire were conducted 2 weeks later to verify the reliability of the CADBS.

### 2.7 Validity analysis

Exploratory factor analysis (EFA) and confirmatory factor analysis (CFA) were performed to analyze the structural validity of the questionnaire. The total sample (*N* = 522) was randomly half-split into two approximately equal subsamples, with one being used for EFA and another for CFA. Prior to performing factor analysis, the Kaiser–Meyer–Olkin (KMO) test (≥ 0.6) and Bartlett’s test of sphericity (*p* < 0.05) were conducted to determine sample adequacy. The scores for the 39 items of the questionnaire were then analyzed using the Varimax method of principal component analysis to explore the factorial pattern. Only factors with an Eigenvalue of > 1 were retained. Factor loadings greater than 0.4 are considered adequate. The factor structure derived from EFA was confirmed in the second half of the sample using CFA to determine if the factor model fit the data from each sample. The structural model fit of the questionnaire was evaluated using the ratio of chi-square value to degrees of freedom (*χ*^2^/*df*), root mean square error of approximation (RMSEA), the comparative fit index (CFI), and the Tucker–Lewis index (TLI). A good fit is indicated by non-significant (*p* ≥ 0.05) *χ*^2^ values. Since this statistic is sensitive to sample size, the other measures were also inspected. For CFA, an RMSEA of ≤ 0.08 was considered an acceptable fit (ideally ≤ 0.05). TLI and CFI values approaching 0.90 (ideally ≥ 0.95) are an indication of good model fit.

### 2.8 Statistical analysis of discrimination

After excluding incomplete samples, the completed questionnaires were entered into EpiData. Continuous variables were expressed as mean ± standard deviation (SD), and discrete variables were expressed as frequency with its proportions. A two-sample t-test or ANOVA test was used to compare scores on different dimensions with different sex, marriage status, age groups, residence, BMI, educational level, and family income. Multivariate linear regression was used to analyze associations between BMI and each appetitive trait, adjusted by age and sex. A significant level of *p*-value < 0.05 was used.

SPSS 25.0 statistical software was used for reliability, validity analysis, and statistical analysis.

## 3 Results

### 3.1 Demographic characteristics of the participants

The analysis of the CADBS was conducted for three rounds. In the first round, the CADBS was initially completed by 168 adults aged between 18 and 58 (Sample 1; see Table 1). Sample 1 had a mean age of 28 ± 6, and a mean BMI of 21.78 ± 3.18. Six months later, the second-round CADBS was completed by a sample of 354 adults aged between 18 and 65 (Sample 2; see Table 1). Sample 2 had a mean age of 29 ± 8 and a mean BMI of 22.10 ± 3.25. A third round contained a sample of 522 adults, who were used (Sample 3; see Table 1) to carry out principal component analysis and confirmatory factor analysis. A sub-sample of 80 participants from Sample 3 (mean age 30 ± 10) completed the CADBS for a second time 2 weeks later, to assess test–retest reliability. Participants from all three rounds were mostly living in urban areas (Sample 1: 76.8%, Sample 2: 69.5%, Sample 3: 78.9%), and the majority of participants were women.

**Table 1:** Demographic information for adults who participated in the evaluation of the Chinese Adults Dietary Behavior Scale (CADBS), 2020–2021.

Group	Sample 1	Sample 2	Sample 3	
	(*n* = 168)	(*n* = 354)	(*n* = 522)	(*n* = 80)
	*n* (%)	*n* (%)	*n* (%)	*n* (%)
Sex				
Men	66 (39.3)	172 (48.6)	229 (43.9)	37 (46.3)
Women	102 (60.7)	182 (51.4)	293 (56.1)	43 (53.7)
Age				
18 to 29	129 (76.8)	245 (69.2)	249 (47.7)	44 (55.0)
30 to 44	34 (20.2)	83 (23.4)	131 (25.1)	26 (32.5)
45+	5 (3.0)	26 (7.4)	142 (27.2)	10 (12.5)
Marriage status				
Married	66 (39.3)	151 (42.7)	295 (56.5)	46 (57.5)
Unmarried	102 (60.7)	203 (57.5)	227 (43.5)	34 (42.5)
Residence				
Urban	129 (76.8)	246 (69.5)	412 (78.9)	59 (73.7)
Rural	39 (23.6)	108 (30.5)	110 (21.1)	21 (26.3)
BMI				
Underweight	22 (13.1)	52 (14.7)	43 (8.2)	13 (16.3)
Normal weight	118 (70.2)	209 (59.0)	335 (64.2)	45 (56.2)
Overweight	28 (16.7)	83 (23.4)	122(23.4)	18 (22.5)
Obesity	4 (2.4)	10 (2.9)	22 (4.2)	4 (5.0)
Education level				
Junior high school degree or below	9 (5.4)	27 (7.7)	44 (8.4)	17 (21.2)
Senior high school degree	11 (6.5)	44 (12.4)	47 (9.0)	13 (16.3)
Bachelor’s degree or above	148 (88.1)	283 (79.9)	431(82.6)	50 (62.5)
Family income*				
≤3,000 CNY/month	48 (28.4)	85 (24.0)	141 (27.0)	42 (52.6)
3,000 ~ 5,000 CNY/month	82 (48.5)	160 (45.1)	156 (29.9)	23 (28.7)
5,000 ~ 10,000 CNY/month	30 (17.7)	88 (24.8)	124 (23.8)	12 (15.0)
>10,000 CNY/month	8 (4.7)	21 (5.9)	101 (19.3)	3 (3.7)

*Exchange rate of CNY 700 to USD is about RMB equal to USD 100.

### 3.2 Item analysis

Data from Sample 1 were used to determine less useful items, resulting in the deletion of 29 items. After deletion, a 47-item questionnaire was given to Sample 2 (containing 354 valid participants) to conduct the same process of item analysis. After two rounds of item analysis, a 39-item questionnaire was finalized with seven dimensions. These dimensions are snacking with eight items, food responsiveness with seven items, emotional eating with five items, restrained eating with five items, food fussiness with four items, healthy dietary awareness with five items, and external eating with five items.

### 3.3 Construct validity

The factor structure derived from EFA was confirmed in the second half of Sample 3. The Kaiser–Meyer–Olkin was 0.87, showing sampling adequacy, and Bartlett’s test confirmed that the factor analysis was appropriate (*p* < 0.001). Seven factors were extracted with eigenvalues greater than 1, and the cumulative contribution of variance accounted for 60.39% of the overall variance. Each remaining item had the highest loading onto a single component and explained the highest variance. Item loadings are presented in Table 2. CFA was used to test the fit of the seven-factor structure derived from EFA. CFA indicators showed acceptable fit in general. The RMSEA was 0.049 (≤ 0.08), and the *χ*^2^/*df* was 1.624, which is less than 3.0. The values of CFI and TLI were 0.903 and 0.892, respectively. The dimensions and items did not change through construct validity for the final version of the questionnaire.

**Table 2:** Factor loadings after varimax rotation of the formal scale for CADBS (39 items).

Dimension name and items	Item source	Loading
Snacking (factor 1; 24.0% variance)		
After work, I like to have some snacks before eating.	Qualitative interview developed	0.75
I like to eat snacks while watching TV or on my phone.		0.73
I cannot eat a meal if I have had a snack just before	AEBQ	0.71
I would eat some snack when I am bored.	Qualitative interview developed	0.66
I like to eat snacks and often prepare some snacks for myself.	Qualitative interview developed	0.64
I will eat snacks at a fixed time every day.	Qualitative interview developed	0.61
I skipped the main meal after snacking before the meal.	Qualitative interview developed	0.60
If I get hungry before bedtime, I will choose to eat snacks.	Qualitative interview developed	0.55
Food responsiveness (factor 2; 9.8% variance)		
Every time I have eaten, I feel like I am not full, and I want to eat something else.	DEBQ	0.73
I can not feel satisfied until I eat full enough.	AEBQ	0.73
I am always thinking about food.	AEBQ	0.69
I look forward to mealtimes.	AEBQ	0.69
If you see others eating, do you also have the desire to eat?	DEBQ	0.67
When I see or smell food that I like, it makes me want to eat.	AEBQ	0.64
Even if I am full, I can still eat a lot of what I like.	DEBQ/AEBQ	0.63
Emotional eating (factor 3; 7.6% variance)		
I eat less when I am anxious	AEBQ	0.81
I eat less when I am angry	AEBQ	0.79
I eat less when I am annoyed	AEBQ	0.79
I eat less when I am upset	AEBQ	0.73
I eat more when I am worried	AEBQ	0.70
Restrained eating (factor 4; 5.7% variance)		
To limit weight, I limit the amount and time spent per meal.	DEBQ	0.84
Do you deliberately eat foods that are slimming?	DEBQ	0.81
When choosing food, I will ignore my taste and choose foods that help me lose weight.	DEBQ	0.75
How often do you refuse food or drink offered because you are concerned about your weight?	DEBQ	0.72
Do you take into account your weight when you eat?	DEBQ	0.61
Food fussiness (factor5; 5.5% variance)		
I would reject similar foods because I did notlike them.	AEBQ	0.80
I see food in a dish that I do not like, so I consciously avoid that dish.	AEBQ	0.80
I reject certain foods because of their taste, appearance, and texture.	AEBQ	0.76
I only eat my favorite foods.	AEBQ	0.68
Healthy dietary awareness (factor 6; 4.8% variance)		
I am more health conscious than before, and often eat foods that I think are beneficial to my health.	Teruel Orthorexia Scale/ Qualitative interview developed	0.74
When cooking, I consciously add some nutritious foods to enhance the nutritional value.	Qualitative interview developed	0.73
After eating fried or spicy food, I will eat more vegetables or fruits as a remedy.	Teruel Orthorexia Scale/ Qualitative interview developed	0.70
I’d rather eat a healthy food that is not very tasty than a good-tasting food that is not healthy	Teruel Orthorexia Scale/ Qualitative interview developed	0.70
I do not want to eat meals that are all meat or vegetarian, because I think they are not nutritious.	Qualitative interview developed	0.65
External eating (factor 7; 3.2% variance)		
If you see or smell something delicious, do you have a desire to eat it?	DEBQ	0.60
When the food changes patterns or the dining environment is more comfortable, I will eat more than usual.	DEBQ	0.60
When having meals with my colleagues and friends, I eat some unhealthy food that I do notusually eat.	Qualitative interview developed	0.54
I will choose food because the media promotes its health.	Qualitative interview developed	0.53
When buying food, I am attracted by the packaging, appearance, and taste of the food.	DEBQ	0.53

The specific definition for each dimension was established to understand the possible meaning of the items in each dimension. Dimension 1 “Snacking” contains eight items, reflecting the respondents’ preference for snacks and motivation to eat snacks during their daily eating activities. Dimension 2 “Food Responsiveness” contains seven items, which indicate the desire to eat food when they see or smell food or are supplied with food. Dimension 3 “Emotional Eating” contains five items that describe the behavior of eating habits in daily life in response to emotions. Dimension 4 “Restrictive Eating” contains five items, reflecting respondents’ restriction of dietary content, concepts, and behaviors. Dimension 5 “Food Fussiness” contains four items, reflecting how picky the participant is toward a variety of foods. Dimension 6 “Healthy Dietary Awareness” contains five items, measuring awareness and behaviors in relation to healthy dietary concepts adopted by individuals. Dimension 7 “External Eating” contains five items, reflecting the vulnerability of eating speed, quantity, and choice of food to external environmental influences.

### 3.4 Reliability analysis

Table 3 shows that the final version of CADBS had adequate internal consistency with Cronbach’s *α* = 0.89. All dimensions of the CADBS had substantial internal consistency with Cronbach’s *α* for each of them ≥ 0.73. The split-half reliability of the questionnaire was 0.92, and for the seven dimensions it ranged from 0.76 to 0.88. Test–retest reliability in the subsample of 80 participants revealed good external reliability, with all values greater than 0.70, except for restrictive eating, healthy dietary awareness, and external eating. Thus, each dimension of CADBS showed acceptable internal consistency with the overall questionnaire.

**Table 3:** Reliability coefficients of the formal scale for CADBS.

Dimension	Cronbach’s α coefficient	Split-half reliability coefficient	Test–retest reliability coefficient
Snacking	0.86	0.78	0.72
Food responsiveness	0.88	0.89	0.73
Emotional eating	0.86	0.88	0.76
Restrained eating	0.83	0.84	0.68
Food fussiness	0.80	0.82	0.74
Healthy dietary awareness	0.77	0.77	0.60
External eating	0.73	0.76	0.60
Total	0.89	0.92	0.74

### 3.5 Content validity

As shown in Table 4, the Pearson’s correlation coefficients among different dimensions ranged from 0.02 to 0.54. The correlation coefficients between each dimension and the overall questionnaire ranged from 0.43 to 0.68. Specifically, the coefficients of snacking, food responsiveness, and external eating were more than 0.6, so they showed a strong correlation with the overall questionnaire, respectively. Snacking was found to be related to food responsiveness, restrained eating, food fussiness, healthy dietary awareness, and external eating. Food fussiness was not significantly associated with healthy dietary awareness, but it was positively correlated with restrained eating.

**Table 4:** Pearson’s correlation coefficients among dimensions of CADBS.

Dimensions	Snacking	Food responsiveness	Emotional eating	Restrained eating	Food fussiness	Healthy dietary awareness	External eating
Snacking	1.00	0.549**	−0.075	−0.190**	−0.254**	−0.253**	0.341**
Food responsiveness		1.00	0.023	−0.174**	−0.217**	−0.303**	0.476**
Emotional eating			1.00	−0.143**	−0.158**	−0.138**	0.215**
Restrained eating				1.00	0.137**	0.355**	−0.144**
Food fussiness					1.00	0.073	−0.408**
Healthy dietary awareness						1.00	−0.329**
External eating							1.00
Total	0.640**	0.659**	0.434**	0.547**	0.558**	0.587**	0.682**

*p<0.05; **p < 0.01.

### 3.6 Statistical analysis of discrimination

The distribution of dietary behavior scores in seven dimensions across demographic information is shown in Table 5. Apart from emotional eating, each dimension showed a significant difference in genders (*p* < 0.05). Excluding healthy eating awareness, each dimension showed a significant difference in age groups (*p* < 0.05). The average score of emotional eating showed a decreasing trend with age, except for the age group of 30–44. For marital status, snacking, food responsiveness, food fussiness, and external eating showed significant differences (*p* < 0.05). In terms of BMI, snacking and restrained eating were significantly different. At all education levels, all five dimensions, except for restrained eating and emotional eating, showed significant differences (*p* < 0.05). With regard to monthly income per caregiver, restrained eating and healthy eating awareness were significantly different (*p* < 0.05).

**Table 5:** Distribution of dietary behavior in Chinese adults (mean±SD).

Group	Snacking	Food responsiveness	Emotional eating	Restrained eating	Food fussiness	Healthy dietary awareness	External Eating
Sex							
Male	2.39 ± 0.70	2.63 ± 0.75	2.97 ± 0.78	2.49 ± 0.89	2.78 ± 0.83	2.81 ± 0.69	2.99 ± 0.62
Female	2.57 ± 0.76^a^	2.85 ± 0.81^a^	2.94 ± 0.81	2.66 ± 0.83^a^	2.99 ± 0.83^a^	3.01 ± 0.75^a^	3.15 ± 0.63^a^
Marriage							
Married	2.26 ± 0.71	2.54 ± 0.73	3.00 ± 0.79	2.62 ± 0.84	2.77 ± 0.83	2.87 ± 0.73	2.97 ± 0.58
Unmarried	2.79 ± 0.66^b^	3.03 ± 0.78^b^	2.89 ± 0.80	2.54 ± 0.88	3.07 ± 0.82^b^	2.99 ± 0.73	3.22 ± 0.67^b^
Age							
18 to 29	2.75 ± 0.68	2.97 ± 0.79	2.90 ± 0.82	2.49 ± 0.86	3.06 ± 0.85	2.91 ± 0.75	3.19 ± 0.64
30 to 44	2.33 ± 0.72^c^	2.61 ± 0.70^c^	3.13 ± 0.76^c^	2.67 ± 0.77	2.83 ± 0.79^c^	2.98 ± 0.67	3.04 ± 0.56^c^
45+	2.18 ± 0.69^c^	2.50 ± 0.77^c^	2.87 ± 0.77^d^	2.68 ± 0.92^c^	2.68 ± 0.81^c^	2.89 ± 0.75	2.91 ± 0.64^c^
Residence							
Urban	2.45 ± 0.72	2.73 ± 0.78	2.97 ± 0.80	2.63 ± 0.86	2.92 ± 0.79	2.96 ± 0.73	3.12 ± 0.61
Rural	2.64 ± 0.79^e^	2.86 ± 0.81	2.89 ± 0.79	2.43 ± 0.83^e^	2.81 ± 0.99	2.78 ± 0.72^e^	2.93 ± 0.69^e^
BMI							
Underweight	3.22 ± 0.81	3.16 ± 0.96	3.10 ± 0.84	2.12 ± 1.04	3.13 ± 0.71	3.04 ± 0.91	2.87 ± 0.73
Normal weight	3.49 ± 0.74^f^	3.25 ± 0.79	3.02 ± 0.79	2.60 ± 0.84^f^	2.90 ± 0.87	2.94 ± 0.70	2.91 ± 0.64
Overweight	3.63 ± 0.68^f^	3.28 ± 0.70	3.05 ± 0.78	2.73 ± 0.77^f^	2.84 ± 0.76	2.86 ± 0.67	2.94 ± 0.54
Obesity	3.72 ± 0.79^f^	3.23 ± 1.00	3.31 ± 0.87	2.55 ± 1.00	2.77 ± 0.98	2.70 ± 1.03	3.06 ± 0.83
Education level							
Junior high school degree or below	2.17 ± 0.76	2.44 ± 0.73	2.91 ± 0.91	2.39 ± 0.89	2.31 ± 0.97	2.61 ± 0.75	2.64 ± 0.78
Senior high school degree	2.45 ± 0.83	2.59 ± 0.80	2.91 ± 0.67	2.40 ± 0.96	2.66 ± 0.78	2.81 ± 0.85	2.95 ± 0.68^g^
Bachelor’s degree or above	2.53 ± 0.72^g^	2.80 ± 0.79^g^	2.96 ± 0.80	2.63 ± 0.84	2.98 ± 0.80^g^^,^^h^	2.97 ± 0.71^g^	3.14 ± 0.59^g^
Family income							
≤3,000 CNY/month	2.56 ± 0.79	2.83 ± 0.89	2.85 ± 0.71	2.44 ± 0.83	2.76 ± 0.99	2.85 ± 0.81	2.98 ± 0.75
3,000 ~ 5,000 CNY/month	2.52 ± 0.75	2.74 ± 0.74	2.98 ± 0.82	2.56 ± 0.89	2.93 ± 0.80	2.85 ± 0.77	3.12 ± 0.62
5,000 ~ 10,000 CNY/month	2.39 ± 0.71	2.79 ± 0.78	2.94 ± 0.76	2.60 ± 0.87	2.98 ± 0.77	2.98 ± 0.65	3.15 ± 0.55
>10,000 CNY/month	2.46 ± 0.68	2.62 ± 0.74	3.07 ± 0.91	2.82 ± 0.79^j^^,^^k^	2.95 ± 0.71	3.07 ± 0.62^j^^,^^k^	3.08 ± 0.56

a *P* < 0.05 vs. male.

b *P* < 0.05 vs. married.

c *P* < 0.05 vs. 18 to 29.

d *P* < 0.05 vs. 30 to 44.

e *P* < 0.05 vs. urban.

f *P* < 0.05 vs. underweight.

g *P* < 0.05 vs. junior high school degree or below.

h *P* < 0.05 vs. senior high school degree.

j *P* < 0.05 vs. ≤3,000 CNY/month.

k *P* < 0.05 vs. 3,000–5,000 CNY/month.

Table 6 presents the relationships between BMI and the seven dietary dimensions, as assessed through correlation and regression analyses. Pearson’s correlation coefficients were calculated for the total sample, with additional stratification by gender and age. Multivariate linear regression was employed, comprising an unadjusted model with seven dimensions as predictors of BMI, and a second model adjusted for gender and age. In the total sample, four dimensions showed significant correlations with BMI. Snacking (*r* = 0.199), restrained eating (*r* = 0.150), and external eating (*r* = 0.100) were positively correlated, while food fussiness (*r* = −0.135) was negatively correlated. Stratified analyses further indicated that the correlation for snacking was significant only in men (*r* = 0.249, 95% CI 0.123 to 0.367) and the 18–29 years age group (*r* = 0.167, 95% CI 0.044 to 0.286), but not in women or older age groups. In the unadjusted regression model (I), when all seven dimensions were included simultaneously, four dimensions showed significant associations with BMI: snacking (*β* = 0.878, *p* < 0.01) and restrained eating (*β* = 0.832, *p* < 0.01) were positively associated, while food fussiness (*β* = −0.404, *p* < 0.05) and healthy dietary awareness (*β* = −0.402, *p* < 0.05) were negatively associated. After adjusting for gender and age in model (II), coefficient estimates fluctuated: snacking (*β* = 0.422, 95% CI 0.039 to 0.806, *p* < 0.05) and restrained eating (*β* = 0.675, 95% CI 0.387 to 0.963, *p* < 0.01) remained positively associated, while food responsiveness emerged as a new positive predictor (*β* = 0.412, 95% CI 0.041 to 0.783, *p* < 0.05), and healthy dietary awareness remained negatively associated (*β* = −0.396, 95% CI –0.748 to −0.045, *p* < 0.05).

**Table 6:** Pearson’s correlation coefficients and multivariate regression analysis between the CADBS and unadjusted and adjusted correlations with BMI.

CADBS	Pearson’s correlation coefficient (95% CI)						Multivariate β (95% CI)	
	Overall sample	Men	Women	18 to 29 years	30 to 44 years	45 + years	Model (I)	Model (II)
Snacking	0.199** (0.115,0.280)	0.249** (0.123,0.367)	0.110 (−0.006,0.221)	0.167** (0.044,0.286)	0.028 (−0.145,0.198)	−0.029 (−0.193,0.137)	0.878** (0.465,1.291)	0.422* (0.039,0.806)
Food responsiveness	−0.084 (−0.169,0.001)	−0.148* (−0.272,-0.019)	0.041 (−0.074,0.155)	−0.026 (−0.150,0.099)	0.059 (−0.144,0.228)	0.074 (−0.092,0.236)	0.174 (−0.236,0.585)	0.412* (0.041,0.783)
Emotional eating	0.042 (−0.044,0.127)	0.122 (−0.008,0.247)	−0.014 (−0.129,0.100)	0.049 (−0.075,0.173)	0.072 (−0.101,0.240)	0.100 (−0.066,0.260)	0.113 (−0.212,0.439)	0.196 (−0.096,0.489)
Restrained eating	0.150** (0.065,0.233)	0.210** (0.083,0.331)	0.173** (0.060,0.282)	0.075 (−0.050,0.197)	0.043 (−0.130,0.213)	0.260** (0.010,0.407)	0.832** (0.517,1.147)	0.675** (0.387,0.963)
Food fussiness	−0.135** (−0.218,-0.050)	−0.118 (−0.244,0.012)	−0.086 (−0.199,0.029)	−0.121 (−0.242,0.003)	−0.101 (−0.268,0.072)	0.061 (−0.105,0.224)	−0.404* (−0.737,-0.071)	−0.137 (−0.440,0.165)
Healthy dietary awareness	−0.065 (−0.150,0.020)	−0.041 (−0.170,0.089)	−0.016 (−0.130,0.099)	−0.069 (−0.192,0.056)	−0.127 (−0.292,0.046)	−0.047 (−0.210,0.119)	−0.402* (−0.792, 0.012)	−0.396* (−0.748,-0.045)
External eating	0.100* (0.014,0.184)	0.058 (−0.073,0.186)	0.066 (−0.049,0.180)	0.074 (−0.051,0.197)	0.093 (−0.080,0.260)	−0.089 (−0.250,0.077)	−0.010 (−0.509,0.489)	−0.090 (−0.538,0.358)

*p<0.05; **p < 0.01.

## 4 Discussion

The goal of this study is to develop the CADBS and assess its effectiveness for the evaluation of dietary behavior specifically aimed at Chinese adults. The finalized CADBS consisted of 7 dimensions with 39 items, which include snacking, food responsiveness, emotional eating, restricted eating, food fussiness, healthy dietary awareness, and external eating. Our study showed that the CADBS had good reliability and construct validity. Therefore, it can be considered a valid, reliable, and multidimensional assessment tool to measure dietary behavior among Chinese adults.

The construction of the CADBS includes a conceptual framework built up based on the literature review, expert interviews, and three rounds of surveys with a large sample size. Several statistical methods were applied to evaluate the questionnaire. The internal consistency reliability was tested using Cronbach’s *α* coefficient. The total Cronbach’s *α* was 0.89, higher than 0.6, which indicates overall good internal consistency. The variation of the reliability coefficients of the instrument was between 0.60 and 0.77, which supported test–retest reliability. In addition, the results of CFA showed acceptable fit in general. χ2/*df* was 1.624 (< 3.0), RMSEA was 0.049 (< 0.08), which indicates that the final version was ideal; CFI and TLI were 0.903 and 0.892, respectively. The correlation coefficients between each dimension and the overall questionnaire ranged from 0.43 to 0.68.

Although the test–retest reliability for certain dimensions falls below the widely accepted threshold of 0.70, we consider such fluctuations to be within an acceptable range. This does not fundamentally undermine the questionnaire’s utility, as similar findings have been reported in other published literature. For example, the Eating Disorder Examination-Questionnaire (EDE-Q) demonstrated an overall test–retest reliability of 0.76, with specific dimensions—subjective bulimic episodes (0.51), objective overeating episodes (0.39), and shape concern (0.66)—showing lower reliability coefficients than those in our study (34). Despite these lower indicators, the EDE-Q remains widely applied and recognized in the field.

Although several questionnaires have been developed to assess adults’ dietary behavior, there are no such tools solely focused on the Chinese adult population. The DEBQ was developed to assess eating behavior in terms of three eating patterns, including emotional eating, restraint eating, and external eating. The AEBQ was adapted from the Child Eating Behavior Questionnaire, assessing a wide range of eating behavior traits aggregated into four food approaches and four food avoidance traits. A validation study of AEBQ measured its reliability and validity in China, but the sample only included university students, which limits the generalizability of its use to the whole adult population (35). Unlike populations from other regions of the world, which normally maintained stable dietary habits over time, Chinese dietary behavior shifted swiftly with rapid economic growth. Hence, adaptations and modifications from previous questionnaires were needed in order to have a more accurate evaluation of adults’ dietary behavior among the Chinese population. In the CADBS, approximately 67% of the items (26 / 39) were adapted from well-validated and published questionnaires such as the DEBQ, the AEBQ, and the Teruel Orthorexia Scale. Apart from that, snacking and healthy dietary awareness were two newly developed dimensions in the CADBS. With an increasing amount of social pressure and workload in recent decades, a considerable large number of Chinese adults tend to eat some snacks instead of having a formal meal to save time during workdays. This phenomenon can be explained by the evaluation of CADBS, for which snacking accounted for 24% variance in factor loadings among all items. Research showed that snacking is one of the top five consumer trends in 2019 and is expected to gain further momentum in the future (36). Snacking now makes up nearly half of all eating occasions and is one of the most profound changes in consumers’ behavior. The “Snacker” dietary pattern may lead to nutritional vulnerability in young adulthood (22). Previous studies concluded that snacking presented no association with weight status (35, 37). Nevertheless, our study showed that overweight and obese adults would be likely to have more intake of snacks (*β* = 0.422, *P* < 0.05). With regard to healthy dietary awareness, both Chinese and Western cultures have rich dietary traditions and wisdom, but healthy concepts and cooking methods have their own characteristics. Many traditional Chinese ingredients contain rich nutrients and unique effects, such as red dates and goji berries, which are considered healthy foods and are distinctly used in China. While Chinese cuisine emphasizes the balanced combination of natural ingredients such as grains, vegetables, and fruits, Western healthy dietary concepts focus on the balance of protein, fat, and carbohydrates (38). Therefore, in this study, healthy dietary awareness was added to the CADBS.

Our study showed that people with higher BMI tend to have less healthy dietary awareness (*β* = −0.396, *p* < 0.05). The negative correlation indicated that overweight/obese people may have a lack of nutrition knowledge or poor self-control. Aleksandra et al. demonstrated that the relationship between restrained eating and body weight was positive (39). Participants with greater BMI (≥ 25 kg/m^2^) displayed greater intensity of restrained eating in comparison to respondents with a BMI lower than 25 kg/m^2^. In our study, the result is consistent with Aleksandra et al. that people with higher BMI would have a willingness to restrict food intake (*β* = 0.675, *P* < 0.01). Our study was consistent with previous studies in general Western samples (15, 40), showing that the responsiveness towards food for overweight/obese people is higher than that of normal weight.

In addition to the relationship between BMI and the seven dimensions in CADBS, the differences in dietary behavior among a variety of demographic populations were also diagnosed. Our results showed that in all six dimensions (except for emotional eating), men and women displayed significantly different dietary behaviors. This result is consistent with the findings of LOFFLER et al. (41) with the use of TFEQ-18 in Germany. Adults with different marital statuses showed significantly different scores in four dimensions, except for emotional eating, restrictive eating, and healthy dietary awareness. Except for healthy dietary awareness, all six dimensions showed significant differences in age groups. For emotional eating, adults aged 31–45 had the highest score, whereas scores for the other five dimensions showed a smoothly decreasing trend with age. This result strongly supported the reality that in China, adults between the ages of 31 and 45 usually encounter the hard situation of “having responsibilities for both parents and children,” so that their psychological pressure and mood swings can be considerably immense. In contrast, adults aged 31–45 in Western countries may live a comfortable and free life, making them emotionally stable.

### 4.1 Limitations

One of the limitations of the study was that the sampling method used was convenience sampling rather than random sampling, resulting in an unbalanced sample size of females and males. Another limitation was that some indicators during the questionnaire validation process only reached an acceptable level, leaving room for improvement in subsequent research. Hence, the effectiveness, acceptability, and universal applicability of the CADBS still need to be further verified in future studies, which will expand the survey areas to different regions to enhance representativeness and expand the sample size to validate the scale.

## 5 Conclusion

To our knowledge, the CADBS is the first instrument to evaluate dietary behavior among Chinese adults. The preliminary statistical analysis suggests that the questionnaire had good reliability, construct validity, and discrimination. Further studies should expand the survey samples to different regions of the Chinese population. More studies are needed to explore how dietary behaviors would affect gaining weight and to identify strategies to prevent overweight/obesity in Chinese adults.

## Data Availability

The raw data supporting the conclusions of this article will be made available by the authors, without undue reservation.
